# Oxygen Saturation Thresholds for Opioid-induced Respiratory Depression: A Systematic Review

**DOI:** 10.5811/westjem.54000

**Published:** 2026-06-28

**Authors:** Akash Manes, Joban Sran, Ivjot Samra, Balkaran Dhaliwal, Adam Soliman, Nitasha Puri

**Affiliations:** * University of British Columbia, Faculty of Medicine, Undergraduate Medical Program, Vancouver, British Columbia, Canada; † University of British Columbia, Faculty of Science, Vancouver, British Columbia, Canada; ‡ University of British Columbia, Faculty of Medicine, Department of Family Practice, Vancouver, British Columbia, Canada; § Fraser Health Authority, Department of Addiction Medicine and Substance Use Services, Surrey, British Columbia, Canada

## Abstract

**Introduction:**

Hypoventilation and hypercapnia are the primary physiological indicators of opioid-induced respiratory depression (OIRD), while hypoxia is typically a later manifestation. However, use of pulse oximetry to measure oxygen saturation (SpO_2_) level remains the most widely available and commonly used monitoring modality in both clinical and community settings, whereas capnography is not routinely accessible. Given this reality, it is important to evaluate hypoxia thresholds reported in the literature to inform practical detection and intervention strategies. Oxygen saturation monitoring may serve as a valuable support tool to help determine when individuals experiencing opioid toxicity require intervention; however, there is limited consensus on SpO_2_ thresholds that indicate OIRD. Our objective in this study was to evaluate existing evidence on SpO_2_ thresholds for OIRD as a physiological marker to incorporate with clinical assessment to potentially prevent opioid-related outcomes such as hypoxemia, hypoxic brain injury, cardiac arrhythmias, and mortality, and to inform monitoring and intervention strategies for future research.

**Methods:**

We electronically searched Ovid MEDLINE, Ovid Embase, PubMed, and grey literature databases for qualitative and quantitative studies published from 2014–2024. We included studies published in English where participants with opioid use disorder (OUD) experienced opioid toxicity and continuous SpO_2_ monitoring in medical settings. Quality was evaluated using the modified Downs and Black checklist. The primary outcome measure was the SpO_2_ level used to quantify opioid-induced respiratory depression.

**Results:**

Of the 2,864 articles screened, 37 underwent full-text review, and 16 were included in data extraction and analysis (total patients, *N* = 12,887). Reported SpO_2_ thresholds ranged from 85–95% (median 92, interquartile range 90–95). In three studies where patients experienced opioid-induced respiratory depression, SpO_2_ levels dropped to a median value of 80%.

**Conclusion:**

Although hypoxia is a late indicator of opioid-induced respiratory depression, SpO_2_ monitoring remains a widely deployed tool for detecting clinically significant deterioration. Reported SpO_2_ thresholds for defining OIRD ranged from 90–95% across studies of “excellent” and “good” quality, underscoring the need for standardized, patient-centered thresholds to guide monitoring and intervention in future research.

## INTRODUCTION

Opioid toxicity refers to the clinical syndrome resulting from excessive opioid exposure, presenting as central nervous system depression, decreased level of consciousness, respiratory depression, cyanosis, and/or miosis.^1^ The most life-threatening feature of opioid toxicity is opioid-induced respiratory depression (OIRD), which is defined as a reduction in the drive to breathe due to opioid action at μ-opioid receptors in the brainstem respiratory centers, leading to hypoventilation, hypercapnia, and potentially apnea and death.^2^–^5^ International opioid use has doubled in prevalence from 26–36 million people in 2010 to 61 million people in 2020 with epidemics currently ongoing in the United States, Canada, North Africa, West Africa, and the Middle East and Southwest Asia.^6^ According to the World Health Organization (WHO), of the 600,000 drug-related deaths in 2019, approximately 480,000 were related to opioids.^7^ In response, a range of harm-reduction strategies have been implemented including supervised consumption sites with immediate access to naloxone, where trained staff can identify and manage OIRD on site, reducing fatalities and hospital utilization.^8^–^11^

### Opioid Use Disorder

Certain factors are associated with an increased risk of opioid toxicity: using opioids while alone; being male; < 25 years of age; being of Indigenous ancestry; having had previous episodes of opioid-induced respiratory depression, not being on any opioid agonist therapy; and having low socioeconomic status. Structural and access-related factors have also been associated with increased risk, including inconsistent drug supply with variable potency, limited availability of supervised consumption services, barriers related to policing practices, housing instability, limited access to regular healthcare, and food insecurity.^12^–^19^

### Opioid Toxicity

Standardized tools including the Glasgow Coma Scale (GCS), Richmond Agitation-Sedation Scale (RASS), and Pasero Opioid-induced Sedation Scale (POSS) may be used to assess sedation or mental status, but they do not reliably reflect the physiological changes of opioid-induced respiratory depression.^20^ The decision to administer naloxone versus providing only ventilatory support in opioid toxicity is guided by the presence and severity of OIRD, with initial management prioritizing support of the airway and breathing.^21^ Naloxone is not administered if the patient is breathing adequately, regardless of mental status.^21^–^22^ For the layperson, especially in a stressful situation, it can be difficult to accurately assess respiratory rate, depth of breathing, and signs of hypoxemia or hypercapnia.

As clinical judgment determines whether someone receives naloxone, there is both over- and undertreatment. Risks of overtreatment of naloxone include precipitated withdrawal, agitation, pulmonary edema, and loss of analgesia in patients with pain; whereas risks of undertreatment include persistent hypoxemia, progression to cardiac arrest, and death.^22^–^24^ In inpatient or emergency department (ED) settings, continuous monitoring (eg, mental status and vitals check, pulse oximetry, end-tidal carbon dioxide) is feasible due to the availability of medical staff, equipment, and protocols for rapid intervention.

**Population Health Research Capsule**

What do we already know about this issue?

*Hypoventilation and hypercapnia signal opioid-induced respiratory depression (OIRD) early; optimal hypoxia thresholds for intervention are unclear*.

What was the research question?

*Is there a consensus on SpO* *_2_* *thresholds that define opioid-induced respiratory depression?*

What was the major finding of the study?

*SpO_2_ thresholds ranged from 85–95%, while patients with OIRD reached a median SpO_2_ of 80%*.

How does this improve population health?

*Clarifying SpO_2_ targets supports safer opioid monitoring in community settings, lowering risk of hypoxic complications and death*.

### Pulse Oximetry

An objective, cost-effective, and user-friendly technology that can assist in monitoring patients with opioid toxicity is pulse oximetry. Monitoring of oxygen saturation (SpO_2_) levels is widely used for its accessibility, portability, standardization, and ease of use by nonclinicians, providing a noninvasive, continuous, and quantitative measure of hypoxemia. It is also less subjective than clinical assessment tools such GCS, RASS, and POSS. Even in hospital, it can detect desaturation events that may be missed by intermittent clinical checks, especially in settings with high patient-to-nurse ratios.^25^ Its low barrier to use and widespread availability make it attractive for both hospital and home monitoring. Despite hypoxia being a late manifestation of opioid-induced respiratory depression, the broad availability of pulse oximetry relative to capnography makes it pragmatic to examine hypoxia thresholds reported in the literature.

### Oxygen Saturation Threshold in Opioid Users to Prevent Hypoxemia and Opioid Toxicity

Despite these limitations, mSpO_2_ is an objective, noninvasive measure that can supplement clinical signs and symptoms to guide intervention in opioid-induced respiratory depression, particularly in community settings. It provides a threshold for hypoxemia, which is directly correlated to patient-important outcomes such as brain injury, cardiac arrhythmia, and death from tissue hypoxia.^26^ Various SpO_2_ thresholds have been proposed in guidelines and the literature; however, there is no consensus on a specific SpO_2_ threshold to guide interventions, and current practice relies on clinical judgment, which can be variable.^27^,^28^ Addressing this knowledge gap regarding the optimal SpO_2_ target for initiating treatment in opioid toxicity is crucial because it directly impacts the balance between overtreatment and undertreatment, both of which carry significant risks for patients. Therefore, our objective in this study was to review SpO_2_ thresholds, defined as oxygen saturation levels indicative of OIRD, to inform harm prevention and guide future research on developing and testing monitoring protocols.

## METHODS

This study is reported using the PRISMA (Preferred Reporting Items for Systematic Reviews and Meta-analyses) guideline.

### Eligibility Criteria

Studies were eligible for inclusion if study participants met the following criteria: 1) ≥ 18 years of age; 2) had opioid use disorder; 3) had been exposed to opioids; 4) were continuously monitored using pulse oximetry or arterial blood gas; 5) and had experienced either opioid toxicity, hypoxemia, OIRD, ventilation, naloxone administration, hospitalization or death; 6) study design consisted of randomized controlled trials (RCT), cohort studies, systematic reviews/meta-analyses, quality improvement, case-control studies, or cross-sectional studies; and 7) the studies were published in English between 2014–2024. We excluded studies if they involved 1) pediatric populations, animal studies, cancer patients, surgical patients, or patients with conditions known to affect oxygenation or respiratory function (eg, diagnosed sleep-disordered breathing); or 2) study designs such as narrative reviews, case reports, commentaries, editorials, or nonpeer-reviewed articles. The study setting (community, inpatient, or outpatient) was not considered an eligibility criterion. We developed eligibility criteria to ensure comprehensive inclusion of all studies that measured SpO_2_ in patients experiencing opioid toxicity, while focusing on clinically relevant, high-risk populations.[Table t1-wjem-27-886]

### Search Strategy

An electronic search of the databases was conducted on July 23, 2024. The databases searched were Pubmed, Ovid MEDLINE, and Ovid Embase inclusive of all articles from January 1, 2014–July 23, 2024. The complete search strategy for Ovid MEDLINE, Ovid Embase, and Pubmed is found in Supplementary Material 1. Additionally, we conducted a comprehensive grey literature search of government and public health agency websites (U.S. Centers for Disease Control and Prevention, Health Canada, WHO, provincial/state health websites), professional society guidelines (American Heart Association, Canadian Association of Emergency Physicians), clinical trial registries (ClinicalTrials.gov), protocols from supervised consumption services and poison control centers, and conference abstracts. We developed the search strategy in consultation with expert librarian staff at the University of British Columbia Faculty of Medicine. The eligible studies were imported into Covidence, which removed duplicates and allowed for independent study selection.

### Study Selection

Two independent and blind reviewers (AM and JS) completed title and abstract screening of each study. Disagreements were solved by a third reviewer. Duplicates were removed using a Covidence tool that generates a reference list and accurately removes identical citations. AM also verified that the duplicates were accurately removed. An analogous process was used for full-text screening.

### Data Extraction

Data was extracted via a standardized data collection form used independently by two reviewers (AM and JS). Discrepancies during data extraction were resolved through verbal discourse among reviewers upon completion of the data extraction stage. The collected data included basic article information (study ID, title, author names, publication date, sponsorship source, country of publication, journal name, and conflict of interest statements) and study-specific information (study design, setting, sample size, participant age, inclusion/exclusion criteria, number of withdrawals and reasons for withdrawal, SpO_2_ threshold used to define respiratory depression, and the proportion of adverse events). For adverse events, we extracted the study-defined criteria and, when reported, the frequency and classification of events.

### Study Assessment

We used the modified Downs and Black checklist to assess the risk of bias and overall quality of the 16 included studies. The checklist is a questionnaire consisting of 27 questions across five categories: reporting; external validity; internal validity; and power (Supplementary Material 2). As per the researchers, scores are split into the following categories: excellent (26–28 points); good (20–25 points); fair (15–19 points), and poor (≤ 14 points). Two researchers (AM, JS) independently assessed the included studies. Disagreements were resolved through verbal discourse between both reviewers. We assessed reporting bias by reviewing each study’s published protocol, if available. If unavailable, the article’s methods section was compared to its results section to ensure all outcomes were reported.

### Synthesis

We synthesized results through a narrative review, with key study characteristics and outcomes summarized in tables. Heterogeneity across studies was explored descriptively, considering differences in study populations, settings, definitions of opioid-induced respiratory depression, and SpO_2_ thresholds. Inter-rater agreement for study selection and data extraction was assessed using the Cohen kappa coefficient (κ).

## RESULTS

### Study Selection

We selected 2,864 papers for abstract screening: 37 studies were selected for full-text review, and 16 were included in this review, as shown in the PRISMA diagram (Figure). We included all studies that met the eligibility criteria. The title and abstract screening stage showed substantial inter-rater agreement (Cohen κ = 0.77).

### Study Characteristics

The SpO_2_ thresholds in the included studies ranged from 85–95% (median 92%, interquartile range [IQR] 90–95). Country-specific differences were minimal, with thresholds in Australia ranging from 85–95% (92%, [88–94]); the United States 92–95% (94% [92–95]); and single studies from Canada, Germany, Iran, Austria, and the United Kingdom falling within 90–95%, likely reflecting local guideline variations. In the ED setting, the median SpO_2_ threshold used to define opioid-induced respiratory depression was 93% (90–93%). In the setting of medically supervised consumption sites, the median SpO_2_ threshold was 90% (89–94%). Both the clinical research unit and respiratory lab settings included a single study each, with thresholds of 95% and 90%, respectively.

Across studies reporting adverse events, the proportion of patients experiencing hypoxemia or related outcomes ranged from 14–40%, with a pooled event proportion of approximately 18% (Table 2). Event rates varied substantially across studies but did not cluster around a consistent SpO_2_ threshold.

Adverse outcome criteria varied across studies; however, all studies consistently included hypoxemia as a primary measure. Other outcomes generally reflected sequelae of low SpO_2_, including oxygen supplementation, naloxone administration, mechanical ventilation, hospitalization, hypoxic brain injury, and death. In addition to SpO_2_ levels during opioid-induced respiratory depression, several studies reported patient-oriented outcomes. Isoardi et al observed that a single 1.6 mg dose of naloxone reversed respiratory depression in 97% of presentations, with 3% requiring repeat doses; no deaths were reported. Nugent et al documented significant improvements in SpO_2_ for patients treated with naloxone (mean change 23.25%); they did not report on adverse events or deaths. Dibz et al also found significant increases in SpO_2_ of > 20% post-administration, with no adverse events reported.

Overall, practice guidance reflected moderate variability in recommended SpO_2_ thresholds but did not identify a universally accepted standard, with most recommendations falling within a narrow range centered around 90% (Table 4).

### Assessment of Quality

Individual score breakdown is available in Table 5. One study was scored as ‘excellent’; 11 were scored as ‘good’; four were scored as ‘fair’; and zero studies were scored as ‘poor.’ Strengths of these studies included clear objectives, outcomes, and reliable analyses. However, external validity and generalizability varied and were not as strong for four of the included studies. Internal validity, including bias and confounding, also varied and was not as strong for four of the studies included.

## DISCUSSION

Every peer-reviewed study included in this systematic review defined the threshold for opioid-induced respiratory depression ≥ 90% except for Roxburgh 2017, which was considered a study of ‘fair’ quality as per the modified Downs and Black checklist. Despite this, our synthesis reveals no true consensus on the optimal SpO_2_ threshold for intervention in OIRD. This lack of agreement is striking given the widespread use of SpO2 measures in both research and practice.[Table t3-wjem-27-886]

Studies and guidelines were found to have a range of SpO_2_ thresholds, typically between 85–95%, with community and supervised consumption settings often using lower thresholds (≤ 85–90%), and hospital or clinical research settings using higher thresholds (92–95%). Lower SpO_2_ thresholds are employed in community settings to balance sensitivity for clinically significant desaturation with the practical need to avoid unnecessary use of scarce resources.^29^ Because these sites often have less frequent monitoring, limited oxygen supplies, and reduced capacity for rapid on-site escalation of care, resource stewardship is prioritized.^29^ However, acute care settings have access to continuous monitoring and immediate access to advanced respiratory support, allowing clinicians to detect and respond rapidly to even mild hypoxemia.^30^ Hospitals can also escalate care quickly if a patient deteriorates, justifying intervention at higher SpO_2_ thresholds.

The heterogeneity in SpO_2_ intervention thresholds identified in this review may reflect differences in patient populations, including comorbidities, as well as variation in opioid tolerance. Comorbid conditions commonly diagnosed in people with opioid use disorder, such as chronic obstructive pulmonary disease, affect normal resting SpO_2_ values and are associated with increased risk of OIRD; hence, they require different thresholds to determine opioid-induced respiratory depression.^31^–^33^ Additionally, in opioid-naive patients, the risk of OIRD at lower doses is higher than in opioid-tolerant individuals due to a lack of tolerance. These individuals require higher SpO_2_ thresholds considering they are more sensitive to opioid effects and rapidly deteriorate compared to individuals with opioid use disorder.^34^

Differences in institutional guidelines also contribute to the heterogeneity among SpO_2_ thresholds. For instance, in Canada two neighboring provinces have different prehospital response protocols to define OIRD and initiate oxygen therapy. The BC Overdose Prevention Services Guidelines state the SpO_2_ threshold as < 90%, whereas Alberta Health Services state the threshold as < 94%.^27^,^28^ Furthermore, consistent with previous studies, our review also found that naloxone is highly effective in reversing hypoxemia and OIRD, producing rapid improvements in SpO_2_ and with deaths being a rare occurrence in controlled settings.^35^,^36^

In outpatient settings, pulse oximetry is the most feasible and widely available method for monitoring oxygenation, given its noninvasive nature, portability, and ease of use. To make optimal use of pulse oximetry as a clinical indicator, it is helpful to define a reference threshold, similar to those set for other vital signs, which also require context-specific interpretation. Despite this, we discovered no consensus on a SpO_2_ threshold. By highlighting this gap, our study defines a clear agenda for future work: the development of standardized reporting practices.

Prospective research studies are needed to find a threshold that will be generalizable to the broader population, while acknowledging that certain subpopulations, such as those with chronic respiratory conditions or altered baseline oxygenation, may need to be excluded. Importantly, even if such a threshold is established, it would complement rather than replace clinical judgment. Airway support and naloxone remain the cornerstones of management for any patient with opioid toxicity and evidence of respiratory compromise. A standardized threshold would, however, be particularly valuable for nonclinicians and in situations of diagnostic uncertainty, where rapid recognition and action can mean the difference between life and death.

## LIMITATIONS

This systematic review is limited by the lack of RCTs addressing declines in SpO_2_ during opioid-induced respiratory depression. This is likely due to ethical constraints in conducting RCTs where patients are experiencing OIRD. This reliance on observational study designs increases susceptibility to bias and limits the strength of inferences as seen in the scoring of non-RCTs in the modified Downs and Black checklist. Another limitation is the heterogeneity of included studies in terms of study setting (eg, emergency department versus supervised consumption sites) and country, which may limit the generalizability of findings across health systems and care contexts. Methodological limitations of this review include the restriction to English-language articles, the exclusion of studies published prior to 2014, and the inclusion of only participants with a diagnosed substance use disorder. While these criteria were selected to focus on clinically relevant data in high-risk populations, they may have excluded potentially informative studies.

Future studies should focus on conducting prospective research using the conservative range of SpO_2_ thresholds to determine feasibility of the threshold, test statistics, prevention of adverse events, and mortality reduction. Future studies should also ethically investigate SpO_2_ thresholds in cases of opioid toxicity to establish clear criteria for early intervention and improved patient outcomes.

## CONCLUSION

Currently, assessing a person with opioid use disorder involves being diligent about the clinical signs and symptoms of opioid-induced respiratory depression. Individuals without healthcare backgrounds have difficulty in recognizing these signs and symptoms. While an oxygen saturation threshold alone would be insufficient to reliably indicate OIRD, it remains an objective measure that can serve as a valuable support tool. Therefore, prospective studies should test SpO_2_ thresholds ranging from 90–95% in relation to opioid-induced respiratory depression to identify a standardized threshold.

## Supplementary Information





## Figures and Tables

**Figure:**
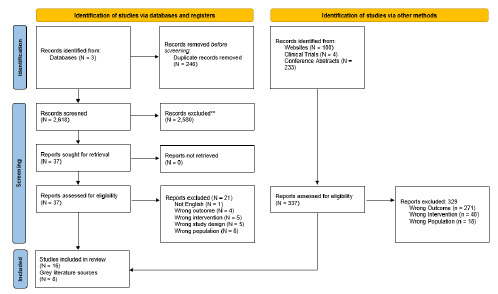
PRISMA flow diagram illustrating the study selection process, including the number of studies screened, excluded, and included in the systematic review. *PRISMA*, Preferred Reporting Items for Systematic Reviews and Meta Analyses.

**Table 1 t1-wjem-27-886:** Study characteristics, including country, study design, and oxygen saturation threshold, stratified by study setting in a review of studies evaluating oxygen saturation targets for opioid poisoning across supervised consumption sites, emergency departments, prehospital emergency medical services, and clinical research settings.

Setting	Article	Country	Article Type	Oxygen Saturation Threshold
Supervision Consumption Site	Roxburgh 2017^37^	Australia	Prospective Cohort Study	≤ 85%
	Moe 2024^38^	Canada	Prospective Cohort Study	≤ 90%
	Jolley 2015^39^	Austria	Prospective Cohort Study	< 90%
	Cho 2022^40^	Canada	Retrospective cohort study	< 91%
	Wishik 2021^41^	USA	Retrospective cohort study	< 95%
	Dietze 2019^42^	Australia	Randomized Controlled Trial	< 95%
	Thiessen 2024^43^	Germany	Prospective Cohort Study	NR (SpO_2_ only reported during use)
Emergency Department	Azar 2020^44^	Iran	Randomized Controlled Trial	< 90%
	Clemency 2019^45^	USA	Prospective Cohort Study	< 92%
	Isoardi 2022^46^	Australia	Prospective Cohort Study	< 93%
	Ryan 2015^47^	USA	Retrospective cohort study	< 94%
	Sabzghabaee 2014^48^	Iran	Randomized Controlled Trial	NR (SpO_2_ only reported at NLX administration)
Prehospital EMS	Isoardi 2023^49^	Australia	Retrospective cohort study	NR (SpO_2_ only reported only at NLX administration)
	Nugent 2019^50^	Canada	Retrospective cohort study	NR (SpO_2_ only reported at NLX administration)
Clinical Respiratory Laboratory	Tas 2022^33^	United Kingdom	Prospective Cohort Study	< 90%
Clinical Research Unit	Lile 2024^51^	Germany	Randomized Controlled Trial	< 95%

*EMS*, emergency medical services; *NLX*, naloxone; *SpO_2_*, oxygen saturation level .

**Table 2:** Opioid formulation and associated adverse effects among patients in studies of opioid-related hypoxemia.

Drug	Paper	SpO_2_ Threshold	Age	Adverse Outcomes Criteria	Reported AE	Sample Size	Proportion
Heroin	Clemency 2019^45^	< 92%	Mean: 34.5	Hypoxemia, O_2_ supplementation, naloxone, hospitalization, death	82	538	0.15
	Dietze 2019^42^	< 95%	Mean: 34	Hypoxemia, naloxone	45	197	0.23
	Isoardi 2023^46^	< 93%	Median: 45	Hypoxemia, naloxone	9	35	0.25
	Jolley 2015^39^	< 90%	Median: 49	Hypoxemia	4	10	0.40
Methadone	Azar 2020^44^	< 90%	Mean: 44	Hypoxemia, mechanical ventilation, hypoxic brain injury, death	13	81	0.16
	Tas 2022^33^	< 90%	Median: 49	Hypoxemia	5	20	0.25
Oxycodone	Roxburgh 2017^37^	≤ 85%	Median: 34	Hypoxemia	1,156	8,382	0.14
Fentanyl	Cho 2022^40^	< 91%	Median: 45	Hypoxemia	743	2,619	0.28
Tramadol	Ryan 2015^47^	< 94%	Median: 41	Hypoxemia, naloxone, mechanical ventilation, death	109	354	0.31
Reported as “Opioids”	Moe 2024^38^	≤ 90%	Mean: 38.6	Hypoxemia, death	77	346	0.22
	Wishik 2021^41^	< 95%	Median: 39	Hypoxemia, O_2_ supplementation, naloxone administration, hospitalization, death	88	305	0.29
Total					2,331	12,887	Mean = 0.24

*AE*, adverse event*; O_2_*, oxygen; *SpO_2_*, oxygen saturation level.

**Table 3 t3-wjem-27-886:** Oxygen saturation levels and interventions in patients experiencing opioid toxicity in a systematic review of published studies.

Study	Extent of Opioid Use	Sample Size	Age (years)	Substance	Oxygen Saturation (%)	Intervention
Isoardi 2022^49^	Opioid Toxicity	197	Median: 41	Heroin	Median: 80 (IQR: 70–90)	Airway and ventilatory support, 1.6 mg naloxone IM (97% single dose, 3% repeat dose)
Nugent 2019^50^	Opioid Toxicity	131	Mean: 41.4	NR	Mean: 79.9 (SD 25.3)	Airway and ventilatory support, 1 mg IN naloxone
Sabzghabaee 2014^48^	Opioid Toxicity	50	Mean: 31.55	Opium	Mean: 72.1 (SD 7.3)	0.4 mg naloxone IV or IN

*IQR*, interquartile range; *IM, intramuscular*; *IN intranasal*; *IV*, intravenous; *NR*, not reported; *SD*, standard deviation.

**Table 4:** Summary of grey literature in a systematic review of studies regarding the optimal oxygen saturation threshold for intervention in opioid-induced respiratory depression.

Title	Source	Year	Setting	SpO_2_ Threshold for Intervention
Practice Guidelines	Ontario Poison Control^52^	2024	In-hospital	90%
	BCCDC^27^	2025	Community	90%
	WHO^53^	2014	Global Guidance	92%
Prehospital clinical protocol	Toward The Heart^54^	2023	Community	90%
	Alberta Health Services^28^	NR	Community	94%
Clinical trial	ClinicalTrials.gov 55	2025	Masimo SafetyNet Alert Technology	85%
Supervised Consumption Site Manual	Vancouver Coastal Health^56^	2024	Community	90%
Clinical Summary	Emergency Care BC^57^	2024	In-hospital	92%

*BCCDC*, British Columbia Centre for Disease Control*; SpO_2_*, oxygen saturation level; *WH*O, World Health Organization.

**Table 5:** Modified Downs and Black checklist scores used to evaluate research quality in a systematic review of studies regarding the optimal oxygen saturation threshold for intervention in opioid-induced respiratory depression.

Study Title	Score (maximum 28)
Buprenorphine to reverse respiratory depression from methadone overdose in opioid-dependent patients- a prospective randomized trial	23
Hospital Observation Upon Reversal (HOUR) with naloxone–a prospective clinical prediction rule validation study	24
Effect of Intranasal vs Intramuscular naloxone on opioid overdose: a randomized clinical trial	27
Dedicated nursing care pathway improved management of opioid-poisoned patients in the emergency department: a before-after observational study	22
Acute opioid withdrawal following intramuscular administration of naloxone 1.6 mg: a prospective out-of-hospital series	19
Understanding heroin overdose: a study of the acute respiratory depressant effects of injected pharmaceutical heroin	18
Physiologic oxygen responses to smoking opioids: an observational study using continuous pulse oximetry at overdose prevention services in British Columbia, Canada.	15
Frequency and severity of non-fatal opioid overdoses among clients attending the Sydney Medically Supervised Injecting Centre.	20
Undetected respiratory depression in people with opioid use disorder	21
Substance consumption and intoxication patterns in a medically supervised overdose prevention program for people experiencing homelessness	21
Tramadol overdose is associated with an increased risk of seizure but not serotonin toxicity	21
A comparison of efficacy of treatment and time to administration of naloxone by BLS and ALS providers.	21
A dose-ranging study of the physiological and self-reported effects of repeated, rapid infusion of remifentanil in people with opioid use disorder and physical dependence on fentanyl.	22
Blood oxygenation and heart rate changes after diamorphine intravenous injection during opioid agonist treatment for outpatients with heroin dependence.	21
Naloxone therapy in opioid overdose patients: intranasal or intravenous? A randomized clinical trial.	20
Opioid overdose and naloxone dosing at insite supervised injection facility in British Columbia: a retrospective cohort study	19
