# Supplementary material for: Oxygen Saturation Thresholds for Opioid-induced Respiratory Depression: A Systematic Review
Source: West J Emerg Med. 2026 Jun 28;27(4):886–94. doi: 10.5811/westjem.54000 (PMC13436640; doi:10.5811/westjem.54000)
Supplement: Supplementary file 2 [file wjem-27-886-s001.docx]

**Supplementary Material 1. Search Strategy for Ovid Medline, Ovid Embase, and PubMed.**

**Ovid Medline Search Strategy**

1. Drug Overdose/
2. exp Opiate Alkaloids/
3. 1 and 2
4. exp narcotic-related disorders/
5. ("opioid disorder*" or "opioid use*" or "opioid overdose*").mp. [mp=title, book title, abstract, original title, name of substance word, subject heading word, floating sub-heading word, keyword heading word, organism supplementary concept word, protocol supplementary concept word, rare disease supplementary concept word, unique identifier, synonyms, population supplementary concept word, anatomy supplementary concept word]
6. ((opioid* or opiate* or fentanyl or heroin or methadone or buprenorphine or oxycodone) adj2 (disorder* or use* or using or usage or overdose* or toxicity or poison*)).mp.
7. ((opioid* or opiate* or fentanyl or heroin or methadone or buprenorphine or oxycodone) adj3 (disorder* or use* or using or usage or overdose* or toxicity or poison*)).mp.
8. 7 not 6
9. 3 or 4
10. 7 or 9
11. Oxygen Saturation/
12. Oximetry/
13. exp Hypoxia/
14. (hypoxia or hypoxemia or spo2 or "o2 saturation" or "oxygen saturation" or "oxygen desaturation" or "oxygen level*" or oximetry).mp. [mp=title, book title, abstract, original title, name of substance word, subject heading word, floating sub-heading word, keyword heading word, organism supplementary concept word, protocol supplementary concept word, rare disease supplementary concept word, unique identifier, synonyms, population supplementary concept word, anatomy supplementary concept word]
15. 11 or 12 or 13 or 14
16. 10 and 15
17. limit 16 to yr="2014 - 2024"

<https://access.ovid.com/custom/redirector/index.html?dest=https://go.openathens.net/redirector/ubc.ca?url=http://ovidsp.ovid.com/ovidweb.cgi?T=JS&NEWS=N&PAGE=main&SHAREDSEARCHID=2HyRALZxwdXfOmcflsjCRT2eOuNEZVLPssfiwnacBbmN6eT8mp8qYuK8WdMHzSJHQ>

**Ovid Embase Search Strategy**

1. drug overdose/
2. exp Opiate Alkaloids/
3. 1 and 2
4. exp narcotic-related disorders/
5. ("opioid disorder*" or "opioid use*" or "opioid overdose*").mp. [mp=title, abstract, heading word, drug trade name, original title, device manufacturer, drug manufacturer, device trade name, keyword heading word, floating subheading word, candidate term word]
6. ((opioid* or opiate* or fentanyl or heroin or methadone or buprenorphine or oxycodone) adj2 (disorder* or use* or using or usage or overdose* or toxicity or poison*)).mp.
7. ((opioid* or opiate* or fentanyl or heroin or methadone or buprenorphine or oxycodone) adj3 (disorder* or use* or using or usage or overdose* or toxicity or poison*)).mp.
8. 7 not 6
9. 3 or 4
10. 7 or 9
11. oxygen saturation/
12. oximetry/
13. exp Hypoxia/
14. (hypoxia or hypoxemia or spo2 or "o2 saturation" or "oxygen saturation" or "oxygen desaturation" or "oxygen level*" or oximetry).mp. [mp=title, abstract, heading word, drug trade name, original title, device manufacturer, drug manufacturer, device trade name, keyword heading word, floating subheading word, candidate term word]
15. 11 or 12 or 13 or 14
16. 10 and 15
17. limit 16 to yr="2014 - 2024"

<https://access.ovid.com/custom/redirector/index.html?dest=https://go.openathens.net/redirector/ubc.ca?url=http://ovidsp.ovid.com/ovidweb.cgi?T=JS&NEWS=N&PAGE=main&SHAREDSEARCHID=7b66i3PEzBe0chDZbtvgOWXtS26hvcIwUDEcnPCp7yIMqK8s2KhG1BTlqFc3ULMIv>

**PubMed Search Strategy**

Filters: from 2014 - 2024

(("Drug Overdose"[Mesh] AND "Opiate Alkaloids"[Mesh]) OR "Narcotic-Related Disorders"[Mesh] OR (("opioid disorder*" OR "opioid use" OR "opioid misuse" OR "opioid overdose*") OR ((opioid* OR opiate* OR fentanyl OR heroin OR methadone OR buprenorphine OR oxycodone) AND (disorder* OR use OR using OR usage OR overdose* OR toxicity OR poison*)) NOT ((opioid* OR opiate* OR fentanyl OR heroin OR methadone OR buprenorphine OR oxycodone) AND (disorder* OR use OR using OR usage OR overdose* OR toxicity OR poison*)))) AND ("Oxygen Saturation"[Mesh] OR "Oximetry"[Mesh] OR "Hypoxia"[Mesh] OR (hypoxia OR hypoxemia OR spo2 OR "o2 saturation" OR "oxygen saturation" OR "oxygen desaturation" OR "oxygen level*" OR oximetry))
